# Induction of cell cycle changes and modulation of apoptogenic/anti-apoptotic and extracellular signaling regulatory protein expression by water extracts of I'm-Yunity™ (PSP)

**DOI:** 10.1186/1472-6882-6-30

**Published:** 2006-09-11

**Authors:** Tze-chen Hsieh, Peili Wu, Spencer Park, Joseph M Wu

**Affiliations:** 1 Department of Biochemistry & Molecular Biology, New York Medical College, Valhalla, NY 10595, USA

## Abstract

**Background:**

I'm-Yunity™ (PSP) is a mushroom extract derived from deep-layer cultivated mycelia of the patented Cov-1 strain of *Coriolus versicolor (CV)*, which contains as its main bioactive ingredient a family of polysaccharo-peptide with heterogeneous charge properties and molecular sizes. I'm-Yunity™ (PSP) is used as a dietary supplement by cancer patients and by individuals diagnosed with various chronic diseases. Laboratory studies have shown that I'm-Yunity™ (PSP) enhances immune functions and also modulates cellular responses to external challenges. Recently, I'm-Yunity™ (PSP) was also reported to exert potent anti-tumorigenic effects, evident by suppression of cell proliferation and induction of apoptosis in malignant cells. We investigate the mechanisms by which I'm-Yunity™ (PSP) elicits these effects.

**Methods:**

Human leukemia HL-60 and U-937 cells were incubated with increasing doses of aqueous extracts of I'm-Yunity™ (PSP). Control and treated cells were harvested at various times and analyzed for changes in: (1) cell proliferation and viability, (2) cell cycle phase transition, (3) induction of apoptosis, (4) expression of cell cycle, apoptogenic/anti-apoptotic, and extracellular regulatory proteins.

**Results:**

Aqueous extracts of I'm-Yunity™ (PSP) inhibited cell proliferation and induced apoptosis in HL-60 and U-937 cells, accompanied by a cell type-dependent disruption of the G_1_/S and G_2_/M phases of cell cycle progression. A more pronounced growth suppression was observed in treated HL-60 cells, which was correlated with time- and dose-dependent down regulation of the retinoblastoma protein Rb, diminution in the expression of anti-apoptotic proteins bcl-2 and survivin, increase in apoptogenic proteins bax and cytochrome c, and cleavage of poly(ADP-ribose) polymerase (PARP) from its native 112-kDa form to the 89-kDa truncated product. Moreover, I'm-Yunity™ (PSP)-treated HL-60 cells also showed a substantial decrease in p65 and to a lesser degree p50 forms of transcription factor NF-κB, which was accompanied by a reduction in the expression of cyclooxygenase 2 (COX2). I'm-Yunity™ (PSP) also elicited an increase in STAT1 (signal transducer and activator of transcription) and correspondingly, decrease in the expression of activated form of ERK (extracellular signal-regulated kinase).

**Conclusion:**

Aqueous extracts of I'm-Yunity™ (PSP) induces cell cycle arrest and alterations in the expression of apoptogenic/anti-apoptotic and extracellular signaling regulatory proteins in human leukemia cells, the net result being suppression of proliferation and increase in apoptosis. These findings may contribute to the reported clinical and overall health effects of I'm-Yunity™ (PSP).

## Background

Throughout history, mushroom and mushroom products have always been revered as food delicacies and are also held in high esteem for their overall health benefits in many cultures, particularly the Orient [1-4]. In East Asian societies, a variety of mushrooms are sold either fresh or as dietary supplements. These products are frequently consumed depending on season of the year as prophylactic measures for common ills and to improve the general well-being of individuals [5]. The notable regard mushrooms are given for promoting wellness of the public at large is perhaps in part attributed to the rather extensive anecdotal and scientific evidence reporting their disease preventive properties, focusing mostly on the potentiation of immune functions and regulation of biological responses [3,4,6]. Beginning in the 1990s, however, it has become increasingly clear that mushrooms, mushroom extracts, and indeed plant/botanical polysaccharides in general, have activities beyond that of the immune system, with suppression of tumorigenesis having the most medical relevance and significance [7-10]. Thus, for instance, polysaccharides with 6-branched 1,3-β glucan structures isolated from the cultured fruit body of edible mushroom *Sparassis crispa *reportedly show antitumor activity when tested against Sarcoma 180 in the ICR strain mice [11,12]. Antineoplastic activity has been demonstrated in polysaccharides isolated from *Pleurotus tuber-regium *[13], and from fruit body of cultivated *Agricus blazei *[14,15]. Several polysaccharide-peptide, and polysaccharide-protein complexes with immunomodulatory and antitumor activities have been isolated and purified from mycelia cultures of *Tricholoma Sp*., an edible mushroom native to Hong Kong [16-18]. Maitake, a mushroom indigenous to northeastern Japan, is recognized as a rich source of polysaccharides with a wide-range of biological and medicinal properties [19,20]. Most notably, gel-purified D-fraction from Maitake characterized as heterogeneous β-(1→6)-branched β-(1→3)-linked alkali-soluble and acid-insoluble polysaccharides [21], show bioactivities spanning the control of immune response, suppression of tumor proliferation, induction of apoptosis, inhibition of metastasis, and regulation of angiogenesis [10,21,22]. Additionally, mushrooms reportedly also contain antitumor proteins capable of inducing apoptosis as well as cell cycle checkpoint arrest in cultured malignant cells [23].

Dietary supplements derived from edible mushroom known as Yunzhi, or *Coriolus versicolor *(*Trametes versicolor*, Fr.) – known as one of six Zhi's recorded in the "Shen Non Compendium Medica" some 2000 years ago – reportedly also show a number of medicinal properties [2,24,25]. Structural and functional analyses of Yunzhi have benefited from the discovery of the patented Cov-1 strain of *Coriolus versicolor *in 1984–1987 by Yang and coworkers, through an exhaustive screen of a large number of strains of Yunzhi [1,4,26]. Subsequently, an innovative industrial scale cultivation method using the mycelia of Cov-1 was developed, which led to the serendipitous discovery, isolation and purification of a family of polysaccharo-peptide, denoted I'm-Yunity™ (PSP) [4,25,27].

I'm-Yunity™ (PSP) has demonstrated potent immunomodulatory and antitumor activities in tissue culture studies, based principally on data using flow cytometry [27-31]. In previous studies, we have observed that ethanol and water extracts of I'm-Yunity™ (PSP) exerted dose- and time-dependent anti-proliferative effects in human promyelocytic HL-60 leukemic cells. Flow cytometric analyses showed that low doses of ethanol and water extracts of I'm-Yunity™ (PSP) induced partial cell arrest in the G_1 _phase, whereas high doses resulted in apoptotic cell death. Moreover, water extracts were found to have significantly more pronounced anti-cellular effects than ethanol extracts [28]. In addition, water and ethanol extracts of I'm-Yunity™ (PSP) markedly increased the secretion of IL-1β and IL-6 with concomitant reduction of IL-8 in HL-60 cells, while having no affect on growth and lymphokine expression in normal human lymphocytes [28]. The molecular details by which I'm-Yunity™ (PSP) exerts its biological effects remain incompletely understood.

To further investigate the mechanism of action of I'm-Yunity™ (PSP), we studied the effects of water extracts of I'm-Yunity™ (PSP) on growth, viability and cell cycle traverse using human HL-60 and U-937 leukemia cells. We found a similar degree of inhibition of cell growth and induction of apoptosis, accompanied by differential targeting of G_1_/S and G_2_/M cell cycle phase transition, respectively, in the HL-60 and U-937 cells. Furthermore, in HL-60 cells, we showed that growth suppression by water extracts of I'm-Yunity™ (PSP) was correlated with time- and dose-dependent inhibition of retinoblastoma protein Rb expression. Correspondingly, induction of apoptosis in I'm-Yunity™ (PSP)-treated cells – evident by cleavage of poly(ADP-ribose) polymerase (PARP) from its native 112-kDa form to the 89-kDa truncated product – was matched by a significant decrease in the expression of anti-apoptotic proteins bcl-2 and survivin, and an increase in apoptogenic proteins bax and cytochrome c. Moreover, HL-60 cells treated with I'm-Yunity™ (PSP) also showed a diminished p65 and p50 forms of transcription factor NF-κB, which paralleled the reduced expression of COX2. We further observed that suppression of HL-60 cell growth was positively correlated with an increase in signal transducer and activator family of transcription factors STAT1, and conversely, with reduction in the expression of activated form of ERK. We postulate that the observed cell cycle and apoptogenic/anti-apoptotic and extracellular signaling regulatory protein expression changes resulting from treatment by water extracts of I'm-Yunity™ (PSP) are mechanistically linked to its reported clinical attributes and general health beneficial effects.

## Methods

### Source of I'm-Yunity™ (PSP)

I'm-Yunity™ (PSP) was supplied by ICM Holdings Ltd. (Hong Kong, China), as a mushroom product produced from deep-layer cultivated mycelia of Cov-1 according to Good Manufacturing Practice (GMP) standards. Briefly, following a 2.5–3 day batch fermentation culture [27], the biomass containing mycelia was extracted with hot water at 95°C for 4–5 h. The aqueous extract was differentially precipitated with ethanol, and fractionated using a proprietary scheme for enriching and retaining bioactive polysaccharo-peptides with molecular weights >40-kDa and an average polysaccharide:peptide ratio of 2:1 [28]. Additional quality control of I'm-Yunity™ (PSP) involved determination of heavy metal contents, microorganism contamination, authentication of elution profiles on high pressure liquid chromatograph, and supplementary bioactivity-guided assays, performed by laboratories at the manufacturing facilities and also independently as coded samples by commercial testing centers.

### Preparation of water extracts of I'm-Yunity™ (PSP)

**Figure 1:**
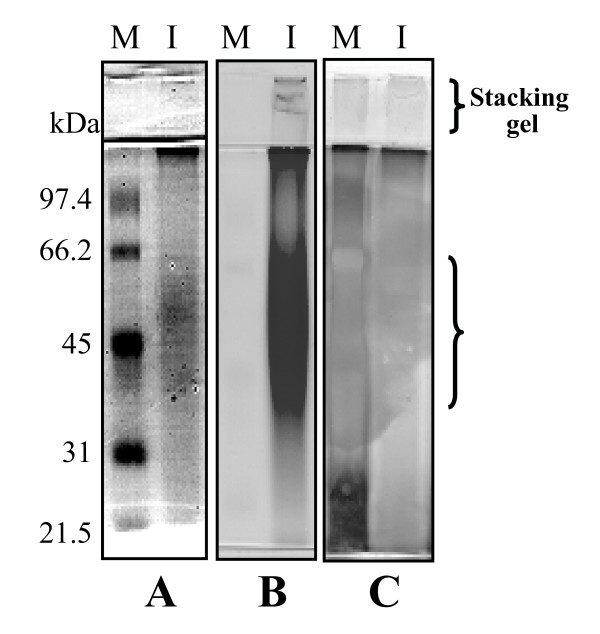
Analysis of constituents of water extracts of I'm-Yunity™ (PSP) by SDS-PAGE and staining with Coomassie blue (***panel A***), Schiff's reagent from Sigma (***panel B***), and silver staining reagent from MP Biomedicals (***panel C***). In each panel, lane M corresponds to the staining pattern of protein markers, with arrows pointing to the position of migration of different molecular weight proteins. Correspondingly, lane I shows the migration of samples of water extracts of I'm-Yunity™ (PSP) after they were separated on 10% SDS-PAGE. The patterns revealed by different stains support the interpretation that I'm-Yunity™ (PSP) is a family of heterogeneous carbohydrate-containing polypeptides.

To prepare water extracts of I'm-Yunity™ (PSP), contents of each capsule (containing 340 mg powder) were suspended in 3.3 ml of water. The suspension was stirred with intermittent mixing at 150 rpm for 60 minutes at room temperature. The insoluble material was removed by centrifugation in a micro-centrifuge. The soluble supernatant was sterilized by passing through a 0.22 μm filter and kept in aliquots at 4°C. Water extracts of I'm-Yunity™ were analyzed by SDS-PAGE followed by staining with Coomassie blue, also using the Schiff's reagent from Sigma (this reagent is modified from the Periodic acid-Schiff (PAS) method and is designed to identify glycoproteins), and with Rapid-Ag-Stain from MP Biomedicals (Figure 1A–C). Staining with Coomassie blue revealed a major and minor band migrating with average molecular weights of 45-kDa and 35-kDa, respectively (Figure 1A). Gels stained with the Schiff's reagent showed a magenta pattern with a very light pink background; the most intensely stain corresponded to a very broad protein band migrating in the vicinity of >45-kDa (Figure 1B). Silver staining did not produce a distinct pattern (Figure 1C). As a whole, these results supported the interpretation that water extracts of I'm-Yunity™ (PSP) contain highly heterogeneous polysaccharo-peptides, with variable carbohydrate moieties linked to different side chain monosaccharides.

### Cell culture and growth inhibition assay

Human HL-60 and U-937 leukemia cells were obtained from the American Type Culture Collection (ATCC, Rockville, MD), and cultured as described [5,28,32-35]. For treatment of either cell type, the stock aqueous extract of I'm-Yunity™ (PSP) was first diluted in tissue culture media and then added to cultured cells to give the final indicated doses. In a typical experiment, 5 ml of cells at a density of 1 × 10^5 ^cells/ml were seeded in T25 flasks. Next, different amounts of aqueous extracts of I'm-Yunity™ (PSP) were added into the culture media. At the specified times, control and treated cells were harvested. Cell count was performed using a hemocytometer and cell viability was determined by trypan blue exclusion [5,28,34,35]. In addition, cell viability of control and treated cells was also measured using the MTT assay [35]. Harvested cells were washed twice with PBS, and pellets were stored at -80°C for additional biochemical and molecular analyses.

### Effects of I'm-Yunity™ (PSP) on cell cycle progression

Cell cycle phase distribution was assayed by flow cytometry. Following a 3-day treatment of HL-60 or U-937 cells with different amounts of water extract of I'm-Yunity™ (PSP) (0.1, 0.5, and 1.0 mg/ml), cells were washed with PBS and stained with 1.0 μg/ml DAPI containing 100 mM NaCl, 2 mM MgCl_2 _and 0.1% Triton X-100 (Sigma) at pH 6.8, as described [28,32,36-38]. The DNA-specific DAPI fluorescence was excited with UV light emitting laser (Ni-Cad), and collected with appropriate filters in an ICP-22 (Ortho Diagnostic, Westwood, MA) flow cytometer. MultiCycle software from Phoenix Flow Systems (San Diego, CA) was used to deconvolute the cellular DNA content histograms to obtain quantitation of the percentage of cells in the respective phases (G_1_, S and G_2_/M) of the cell cycle. Flow cytometry was also used to show cells undergoing apoptosis, evident by the appearance of the sub-G_1 _peak [32,39].

### RNA isolation and quantitative real-time PCR of cyclooxygenase 2 (COX2)

COX2 mRNA expression was assayed using semi-quantitative RT-PCR or quantitative real-time PCR [34,40-42]. Total cellular RNA was isolated from day 3 control and I'm-Yunity™ (PSP) treated HL-60 cells using TRIzol reagent (InVitrogen) according to protocols provided by the manufacturer. RNA purity and quantitation was determined by agarose gel electrophoresis and A_260/280 _absorbance ratio. First strand cDNA synthesis used 2 μl total RNA incubated at 42°C for 50 min with Superscript RNase H^- ^reverse transcriptase (InVitrogen). A 10-fold diluted cDNA served as the template for PCR. To amply and quantitate the human COX2 gene, PCR was performed using the Lightcycler PCR system (Roche Diagnostics, Mannheim, Germany) in glass capillaries in a final volume of 20 μl with addition of 1× Lightcycler master mix, 3 mM MgCl_2_, 1 μM of COX2 primer sets (sense 5'-ATG GGG TGA TGA GCA GTT GT-3' and antisense 5'-TGA GGC AGT GTT GAT GAT TTG-3'), and 2 μl of cDNA template or external standard template. The amplification reaction consisted of heating samples at 95°C for 30 s followed by 40 cycles of heating at 20°C/s to 95°C with a 1-s hold, cooling at 20°C/s to 55°C with a 1-s hold, and heating at 20°C/s to 72°C with a 10-s hold. A melting curve was generated by heating the product at 20°C/s to 95°C, cooling it at 20°C/s to 60°C, and slowly heating at 0.2°C/s to 95°C, with fluorescence collection at 0.2°C intervals.

Human COX2 was amplified by conventional PCR and cloned into PCR 2.1-TOPO vector (InVitrogen) for transformation in TOP10 competent cells. Plasmid DNA containing human COX2 was purified using the Mini Plasmid Extraction Kit (Qiagen, Valencia, CA). Real-time PCR was performed using the Light Cycler DNA Master SYBR Green 1 kit according to manufacturer's instructions. Ten fold serial dilutions of the purified plasmid DNA containing 100 to 10^7 ^copies of COX2 in 2 μl were used as standards to calculate COX2 mRNA copy number, which is expressed as % of control. The data obtained were analyzed using the Lightcycler software provided by the manufacturer. Only log-linear portion of amplification was chosen for analysis. Experiments were performed in duplicate.

### Preparation of whole cell extracts and western blot analysis

Cells were suspended in buffer (50 μl/10^6 ^cells) containing 50 mM Tris-HCl, pH 7.4, 150 mM NaCl, 1% Triton X-100, 1% sodium deoxycholate, 0.1% SDS, 1 mM EDTA, 1 mM PMSF, 5 μg/ml each of aprotinin, pepstatin, leupeptin, and lysed by 3 freeze/thaw cycles [33,42,43]. The extracts were centrifuged and the clear supernatants were stored in aliquots at -70°C. Ten to 20 μg of proteins were separated on 10% SDS-PAGE. Membranes were probed for expression of Rb, poly(ADP-ribose) polymerase (PARP), bcl-2, survivin, bax, cytochrome c, p50 and p65 forms of NF-κB, STAT1, ERK, and β-actin. Commercial antibodies (from Santa Cruz Biotechnology, Inc.) were used at a dilution of 1:1000. Immunoreactivity was demonstrated by enhanced chemiluminescence (ECL) or color reaction, using the manufacturer's protocol (Kirkegared & Perry Laboratories).

### Preparation of subcellular fractions from control and treated HL-60 cells

Cytosolic and mitochondria fractions were obtained using the Mitochondria isolation kit from Sigma. Harvested control and treated cells washed with ice-cold PBS were suspended in extraction buffer containing 10 mM Hepes, pH 7.5 supplemented with 200 mM mannitol, 70 mM sucrose, 1 mM EGTA, and 0.5 mg/ml BSA. The cell suspension was homogenized by 30 passages through a 25-gauge needle. The homogenate was centrifuged at 600 × g, 5 min and the supernatant transferred to a fresh tube. Further centrifugation of the supernatant at 11,000 × g, 10 min yielded a cytosolic supernatant and a mitochondria pellet. The pellet was resuspended in extraction buffer and centrifuged at 11,000 × g, 10 min. The mitochondria pellet was resuspended in 10 mM Hepes, pH 7.4 containing 250 mM sucrose, 1 mM ATP, 0.08 mM ADP, 5 mM sodium succinate, 2 mM K_2_HPO_4_, and 1 mM dithiothreitol. Efficiency of separation of cytosolic and mitochondrial fractions was confirmed by gel electrophoresis and immunoblotting using the mitochondria specific cytochrome c oxidase antibody (from Molecular Probes).

## Results

### Control of proliferation and cell cycle progression by I'm-Yunity™ (PSP) in HL-60 and U-937 cells

**Figure 2:**
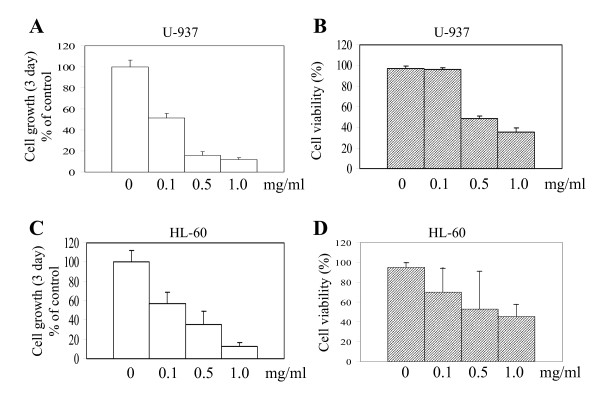
Control of cell growth and viability in U-937 and HL-60 cells by water extracts of I'm-Yunity™ (PSP). Both cell types were cultured and treated with different dose of I'm-Yunity™ (PSP) as described in Materials and Methods. ***Panels A and C ***show suppression of proliferation of U-937 (panel A) and HL-60 (panel C) cells by increasing concentrations of I'm-Yunity™ (PSP). ***Panels B and D ***show reduction of cell viability in U-937 (panel B) and HL-60 (panel D) by I'm-Yunity™ (PSP). Results in panels A-D represent average ± SD of 3–4 experiments.

In previous studies, we demonstrated in HL-60 cells that aqueous extracts of I'm-Yunity™ (PSP) exerted more potent growth inhibitory and apoptosis-inducing effects, compared to 70% ethanolic extracts [28]. Moreover, the reduction in proliferation was accompanied by accumulation of cells in G_1 _simultaneous with a reduction in proportion of cells in the S phase [28]. To investigate whether the growth inhibitory effects of aqueous extracts of I'm-Yunity™ (PSP) can be extended to other myeloid leukemia cells, increasing concentrations of water extracts was added to U-937 cells. A 72 h treatment resulted in significant inhibition of cell proliferation (Figure 2A). Time course studies showed that suppression of growth was evident as early as 24 h after treatment (data not shown). Furthermore, suppression of proliferation was accompanied by a decrease in cell viability using high but not low doses (Figure 2B) of water extracts of I'm-Yunity™ (PSP), suggesting that the polysaccharo-peptide exerts complex anti-cellular effects in the U-937 cells. Similar to our previous observations [28], treatment of HL-60 cells with the same dose range of water extracts of I'm-Yunity™ (PSP) was accompanied by a pronounced inhibition of cell growth and viability (Figure 2C and 2D).

**Figure 3:**
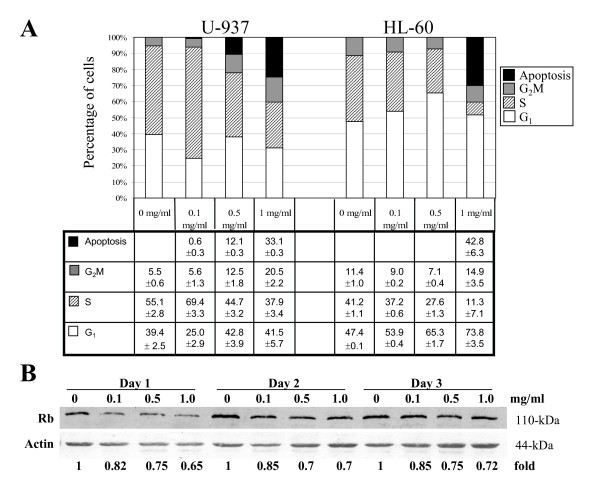
Effects of water extracts of I'm-Yunity™ (PSP) on cellular DNA content frequency histograms showing the cell cycle phase distribution changes and induction of apoptosis in U-937 and HL-60 cells treated for 3 days of treatment with 0, 0.1, 0.5 and 1.0 mg/ml I'm-Yunity™ (PSP) are presented in ***panel A***. Flow cytometric analysis was performed as described in Materials and Methods and results shown represent average ± SD of 2 separate experiments. ***Panel B***. Effects of I'm-Yunity™ (PSP) on Rb expression in treated HL-60 cells. Relative changes in expression of Rb in control and days 1–3 treated cells were determined by Western blot analysis as described in Materials and Methods. The intensity of the Rb and actin immunoreactive bands was quantitated by densitometry and the actin-adjusted Rb expression levels are presented as fold differences, with the control value for each day of treatment showing as 1.

To explore further the manner by which extracts of I'm-Yunity™ (PSP) affected cell growth in these two cell types, flow cytometric analysis was performed and the results illustrated in Figure 3A. In U-937 cells, low dose (0.1 mg/ml) induced an accumulation of cells in the S phase, concomitant with a decrease in proportion of cells in the G_1 _phase (Figure 3A). At higher concentrations (0.5 and 1.0 mg/ml), however, a reduction in S phase, together with an increase in G_2_M phases of the cell cycle was observed. These results provide further support for the notion that a complex mechanism underlies the interaction between extracts of I'm-Yunity™ (PSP) and U-937 cells. In HL-60 cells, however, a dose-dependent increase in G_1_, accompanied by a marked reduction in S phase of the cell cycle resulted from treatment with water extracts of I'm-Yunity™ (PSP) (Figure 3A). These results raise the possibility that water extracts of I'm-Yunity™ (PSP) target both cell cycle checkpoints, as a likely contributing factor for its anti-proliferative activities in tumor cells. To gain further insights on how water extracts of I'm-Yunity™ (PSP) might trigger G_1_/S cell cycle arrest in HL-60 cells, we determined the expression of the retinoblastoma gene product Rb, known to play a pivotal role in controlling the G_1_/S phase transition via a cyclin kinase-mediated, switch on/off mechanism of the binding of transcription factor E2F [33,43,44]. Immunoblot analysis showed a dose-dependent inhibition of Rb expression in treated cells (Figure 3B). The extent of reduction of Rb was correlated with the observed cell cycle changes, suggesting that the attenuation of Rb expression likely plays a critical role in restricting cell progression from G_1 _to S phase, in HL-60 cells.

### Induction of apoptosis in I'm-Yunity™ (PSP)-treated HL-60 cells

**Figure 4:**
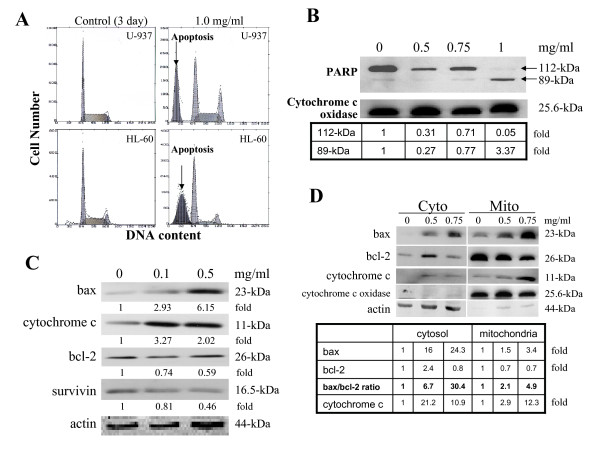
Changes in cell cycle phase distribution and apoptogenic/anti-apoptotic regulatory proteins in control and I'm-Yunity™ (PSP)-treated HL-60 cells. ***Panel A***. Cellular DNA content frequency histograms showing the cell cycle phase distribution changes of U-937 and HL-60 cells following 3 day treatment with 1.0 mg/ml doses of I'm-Yunity™ (PSP). Flow cytometric analysis was performed as described in Materials and Methods. Induction of apoptosis in treated cells is illustrated by cells showing fractional DNA content. ***Panels B-C***, changes in apoptogenic/anti-apoptotic proteins in control and 3 day I'm-Yunity™ (PSP)-treated HL-60 cells. Expression of bax, cytochrome c, bcl-2, survivin, and PARP, identified by their respective molecular weights, was determined by immunoblot analysis, then adjusted for protein loading using cytochrome c oxidase in panel B, or actin in panel C, and presented as fold differences, with the control value showing as 1. ***Panel D***, changes in bax, bcl-2 and cytochrome c in the cytosolic and mitochondria fractions separated using the procedure detailed in Materials and Methods. Expression of the various proteins was adjusted for loading using either cytochrome c oxidase (for mitochondria fraction) or actin (for cytosolic fraction) and presented as fold differences, with the control value showing as 1. The adjusted bax and bcl-2 values were used to calculate the ratio of bax/bcl-2 expression.

Treatment with high dose (1.0 mg/ml) of water extracts of I'm-Yunity™ (PSP) for 3 days also resulted in the induction of apoptosis in both U-937 and HL-60 cells, as evident by the appearance of cells displaying fractional DNA contents in flow cytometric analysis (Figure 4A), and further supported by the cleavage of DNA repair enzyme PARP from its native 112-kDa form to an inactive 89-kDd product (Figure 4B). Since disruption of the mitochondria, accompanied by changes in the relative subcellular distribution of apoptogenic and anti-apoptotic regulatory proteins, has been suggested as being among the key events that occur during apoptosis [37,45-50], we analyzed in detail changes in expression of apoptogenic and anti-apoptotic proteins, notably, bax, cytochrome c, survivin, and bcl-2. Figure 4C shows composite changes in these proteins in day 3, 0.1 and 0.5 mg/ml I'm-Yunity™ (PSP)-treated whole cell lysates. A pronounced increase in bax and cytochrome c was clearly observed, which corresponded to significant decreases in levels of bcl-2 and survivin. As additional proof of the observed protein changes, we analyzed bax, bcl-2 and cytochrome c in the mitochondria and cytosolic fractions. Figure 4D shows dose-dependent increases in the expression of bax in mitochondria and correspondingly cytochrome c in the cytosol; purity of separation into the respective subcellular compartments was supported by the distinctive presence of cytochrome c oxidase in the mitochondria and its absence in the cytosolic fraction. Induction of apoptosis is further demonstrated by dose-dependent I'm-Yunity™ (PSP)-elicited increase in ratio of bax/bcl-2 (Figure 4D). Dose-dependent increase of bax and correspondingly, reduction in bcl-2 expression can be detected as early as 30–36 hours after treatment (data not shown).

### Down-regulation of NF-κB by water extracts of I'm-Yunity™ (PSP)

**Figure 5:**
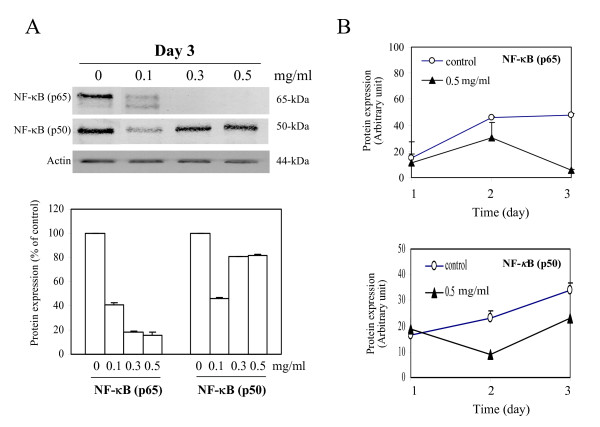
Regulation of expression of NF-κB in HL-60 cells by water extracts of I'm-Yunity™ (PSP). Control and 3 day I'm-Yunity™ (PSP) treated cells were harvested and total protein extracts were prepared, separated by SDS-PAGE and analyzed for expression of NF-κB p65, p50 and actin by immunoblot analysis (***panel A***). Time-dependent changes in actin-adjusted, NF-κB expression by water extracts of I'm-Yunity™ (PSP) are shown in panel B.

NF-κB is a transcription factor that has been implicated in the control of cell growth [51-53]. Constitutive activation of the NF-κB family of transcription factors in various human tumors has been hypothesized to contribute to the development and/or progression of malignancy by regulating the expression of gene sets involved in cell growth and proliferation, circumvention of apoptosis, and the induction of angiogenesis and metastasis [52,53]. We therefore measured changes in the steady state level of NF-κB in HL-60 cells treated with water extracts of I'm-Yunity™ (PSP). Pronounced decreases in p65 form and to a lesser degree also p50 form of NF-κB was found in I'm-Yunity™ (PSP)-treated HL-60 cells in day 3, varying doses of I'm-Yunity™ (PSP)-treated cells (Figure 5A). Figure 5B showed the quantitive determination of changes in both p65 and p50 forms of NF-κB using a single dose of 0.5 mg/ml water extracts of I'm-Yunity™ (PSP).

**Figure 6:**
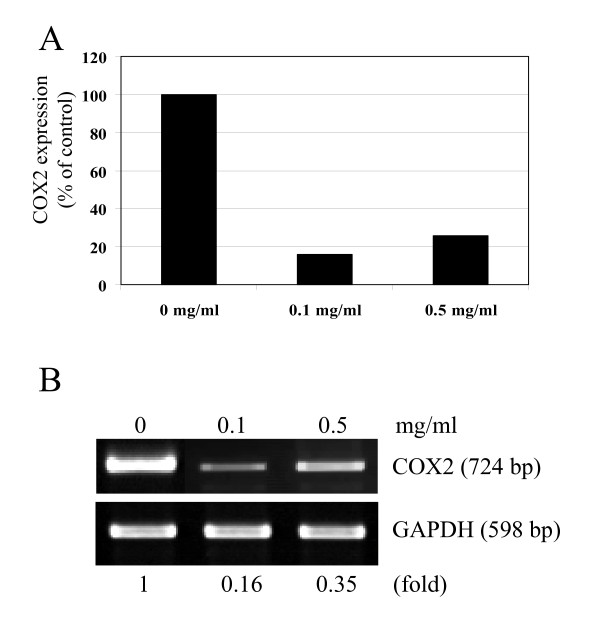
Suppressed expression of COX2 in HL-60 cells by water extracts of I'm-Yunity™ (PSP). Control and treated cells were harvested and expression of COX2 was determined quantitatively by real-time PCR (panel A) and semi-quantitatively by RT-PCR (panel B). Semi-quantitative expression of COX2 in panel B was also adjusted using expression of GAPDH and presented as fold differences, with 1 representing the value in control cells.

NF-κB is involved in transcriptional control of cyclooxygenase 2 (COX2) [54-57]. Therefore, to obtain information on functional consequences associated with the suppression of NF-κB by I'm-Yunity™ (PSP), we determined COX2 expression using quantitative real-time and semi-quantitative RT-PCR. Results in Figure 6 showed that HL-60 cells treated with water extracts of I'm-Yunity™ (PSP) were accompanied by a 65–85% reduction in COX2 expression, depending on the assays used.

### Modulation of extracellular signaling protein expression by I'm-Yunity™ (PSP)

Survival of cells in multicellular organisms is inextricably linked to and dependent on cues from extracellular growth factors. Availability of growth factors and signaling events they elicit contribute to the homeostatic balance between cell proliferation and death. In tumor cells, their endowment with oncogenes together with the hyperactive status of oncogene products often result in amplified and/or persistently activated growth and survival pathways.

Cultured tumor cells incubated with extracts of I'm-Yunity™ (PSP) are accompanied by significant changes in the expression of several important growth modulators and cytokines [28]. Similarly, patients treated with I'm-Yunity™ (PSP) showed increases in interferon-γ expression, a cytokine known to have profound immune regulatory properties [25]. Conceivably, interferon-γ could contribute to the suppression of tumorigenesis indirectly, through modulation of the immune system, as well as directly by exerting anti-proliferative and pro-apoptotic effects. Both mechanisms, in principle, may invoke regulation of gene expression mediated through the STAT (signal transducer and activator of transcription) proteins [58-62]. Recent studies have shown, for example, that STAT1, as a member of the STAT protein family, plays a vital role in the control of apoptosis; namely, lymphocytes from STAT1-/- mice failed to undergo apoptosis, had reduced cytokine processing and apoptogenic proteins caspase 1 and 11, and demonstrated increased proliferation *in vitro*, compared to cells from wild-type animals [63].

**Figure 7:**
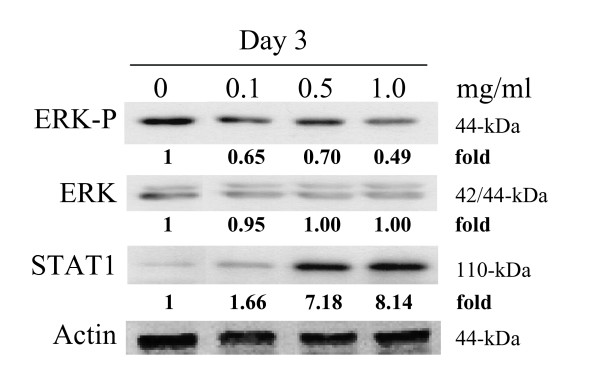
Relative changes in STAT1 and ERK levels in I'm-Yunity™ (PSP)-treated HL-60 cells. Control and treated cells were harvested and total protein extracts were prepared, separated by SDS-PAGE and analyzed for expression of STAT1 and ERK by western blot analysis, with actin expression used to adjust for protein loading.

It is therefore of interest to determine whether treatment of HL-60 cells with water extract of I'm-Yunity™ (PSP) may lead to changes in STAT1. Immunoblot analysis showed a dose-dependent STAT1 increase in day 3, I'm-Yunity™ (PSP)-treated cells (Figure 7). In contrast, analysis of another prominent survival pathway, mediated by the extracellular signal-regulated kinase (ERK), showed that treatment with I'm-Yunity™ (PSP) reduced levels of activated, phosphorylated ERK, while total ERK was unchanged, compared to control cells (Figure 7).

## Discussion

A significant percentage of the estimated two million deaths recorded in the United States each year are attributable to cancer and its ensuing complications. Cancer presents clinically as a constellation of diseases and at all stages – from primary prevention to treatment – can be profoundly and significantly affected by nutrition and diets. The existence of such a link is well supported by decades of epidemiological studies showing that the consumption of plant-based diets is directly associated with reduced risk of cancer [64-76]. The impact of nutrition and diets in prevention of carcinogenesis is formally conceptualized in chemoprevention by Sporn and others in the 1970s, which emphasizes the use of pharmacologic or natural agents to inhibit development or progression of invasive cancer [67-69,71,72,74,77-79]. Research in recent years has begun to focus on "bioactive compounds" in diets with chemopreventive attributes and delineation of their mechanism(s) [67,70,72].

Efficacy of chemopreventive agents may have multiple mechanistic bases: reversal of abnormal differentiation, suppression of cell replication/growth, and induction of apoptosis. Additionally, inhibition of carcinogen activation and increase in their removal by detoxification enzymes are also considered prudent cancer prevention strategies [71,72,74]. Since many of the very same chemopreventive attributes are found in I'm-Yunity™ (PSP), we propose that it, as an example of mushroom and mushroom products, may be included in this category of dietary agents. Indeed, based results from this communication and earlier studies, it may be suggested that I'm-Yunity™ (PSP) is an easily compliant adjunctive treatment particularly germane to human leukemia and other blood-borne hematologic malignancies. Since polysaccharide moieties predominate in I'm-Yunity™ (PSP), it is possible that less chemo- and radio-resistant clones will result from treatment using such an agent.

**Figure 8:**
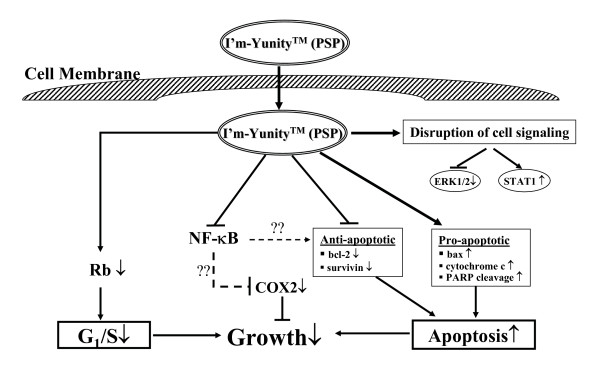
Proposed mechanism of action of I'm-Yunity™ (PSP). In this model, the ability of water extracts of I'm-Yunity™ (PSP) to inhibit cell proliferation and induce apoptosis in cancer cells is hypothesized to involve multiple effects, including 1) disruption of cell cycle control machinery, 2) perturbation in apoptogenic/anti-apoptotic regulatory protein expression promoting the induction of apoptosis, 3) alteration in mitogenic signaling pathways, 4) regulation of gene expression, exemplified by suppression of cell survival transcription factor NF-κB.

The cellular changes reported in this communication as a result of treatment with water extracts of I'm-Yunity™ (PSP) can be explained by the multiple effects it elicits, which include suppression of the retinoblastoma tumor suppressor protein Rb, increased release of cytochrome c from mitochondria to the cytosol, reduced expression of anti-apoptotic protein bcl-2 and survivin, and simultaneously, increase in bax, as well as a disruption in cell signaling events, as collectively depicted in the scheme in Figure 8.

A number of new molecular targets and mechanistic insights have surfaced from the present studies. First and foremost is the observation that I'm-Yunity™ (PSP) down-regulates the expression of NF-κB in HL-60 cells, thereby identifying a new, novel molecular target of this family of bioactive polysaccharo-peptides. It is noteworthy that the effect of I'm-Yunity™ (PSP) on NF-κB correlated well with the suppression of growth, suggesting a causal relationship. NF-κB is a transcription factor known to be functionally associated with cell survival; its constitutive activation in various human malignancies contributes to tumor transformation, development, and progression. These effects may relate to the ability of NF-κB to control the transcription of genes involved in cell growth and proliferation, suppression or escape of apoptosis, acquisition of new vasculature and blood supply, and dissemination to distant location for further growth and colony formation [52-56]. Treatment of HL-60 cells with water extract of I'm-Yunity™ (PSP) caused a precipitous reduction in p65 and to a lesser degree also p50 subunits of NF-κB (Figure 5). This finding is significant since it proffers the notion that this family of polysaccharo-peptides acts in part by forestalling a pivotal factor required for the survival and propagation of tumor cells. The more pronounced decline in p65 relative to p50 may relate to the fact that these two subunits of NF-κB are known to be different gene products and not necessarily regulated in a coordinate fashion. Studies have shown, for example, that p50 is proteolytically derived from a p105 precursor and hence, in principle, could be subjected to more complex mechanism of control, compared to the p65 subunit. Taking these into consideration, it is entirely possible that the p65 and p50 subunits are differentially regulated depending on the dose of I'm-Yunity™ (PSP) used for treating the cells. Conceivably, therefore, low rather than high doses of I'm-Yunity™ (PSP) are effective in suppressing the expression of the p50 subunits. In contrast, a broader dose range of I'm-Yunity™ (PSP) affects the expression of the p65 subunit. It should also be pointed out that our findings do not necessarily contradict the role of I'm-Yunity™ (PSP) as an immune modulator, and similarly, other data pointing to NF-κB as a tumor suppressor rather than a promoter of tumorigenesis [52,54]. It may well be that in normal immune cells, I'm-Yunity™ (PSP) at an appropriate dose range does indeed increases the expression of NF-κB and thereby promotes the increased transcription of genes in immune cells conducive to a heightened immune status. The same cell type consideration may equally apply to recent evidence showing that NF-κB can function as a tumor suppressor by actively repressing anti-apoptotic genes. The acquisition of a non-conventional role for NF-κB, i.e., functioning in tumor suppression rather than promotion, may also depend on stimuli impinging on cells, and just as plausible, the co-expression of genes acting in concert with NF-κB to facilitate transcriptional repressor of anti-apoptotic genes. It has been demonstrated, for example, that ARF (ADP-ribosylation factor) and p53 as well as certain inducers of NF-κB DNA-binding activity, such as ultraviolet light and some chemotherapeutic compounds, can induce the association of NF-κB subunits with transcriptional corepressor complexes, allowing them to function as repressors rather than activators of gene expression [80-82]. Whether these considerations can apply to HL-60 cells, known to have p53-null status but do express the ARF [83-89], in the context of cellular effects elicited by I'm-Yunity™ (PSP) remain to be studied. Further research will also focus on investigation of detailed changes in steady state levels and subcellular distribution of NF-κB, using control and treated cells harboring a stably transfected dominant-negative IκB gene in which amino acid serine at positions 32 and 34 of IκB is mutated to amino acid alanine. These changes should exclude its phosphorylation by IκB kinase, which is required for the degradation of IκB, coincident with the cytoplasm-to-nucleus translocation of NF-κB [51,90,91].

A second new finding revealed in the present studies is the ability of I'm-Yunity™ (PSP) to down regulate COX2 expression. Persistent chronic inflammation plays an important role in carcinogenesis [92-94]. In the early phase of inflammation, COX2 facilitates the production of inflammatory prostaglandins. COX2 has been shown to be overexpressed in a variety of cancers [95-97]. Further, there is compelling evidence from *in vitro *experimental studies that inhibition of COX2 decreases cellular proliferation, increases apoptosis, and modulates genes involved in cell cycle regulation [93,94]. Therefore we measured COX2 expression in control and treated cells. Results in Figure 6 show that COX2 expression was significantly suppressed by treatment with water extracts of I'm-Yunity™ (PSP).

A third important observation relates to the modulation of potent signaling molecules by I'm-Yunity™ (PSP), as exemplified by an increase the expression of STAT1 in parallel with the suppression of the phosphorylated form of ERK (Figure 7). STATs and their protein partners JAKs (Janus kinases) are evolutionarily conserved signaling and cellular communication molecules that enable cytokines to mount a repertoire of host defenses and immunological responses. Similarly, ability of cells to respond to extracellular challenges also depend upon signaling pathways governed by the MAP kinase modules, with sequentially acting protein kinases including ERK as the archetypal form. In both examples, interaction of ligand or an external challenge with membrane-associated receptors are considered to be key initiation events. Because of the importance of the STAT and ERK signaling pathways in homeostatic control of cells, as discussed, it is of interest and pertinent to ask how they might be affected by I'm-Yunity™ (PSP). One possibility is that I'm-Yunity™ (PSP), having structural similarity to proteoglycans [4], act directly as agonists/antagonists of specific surface receptor ligands, and in turn, augment the trigger of the JAK/STAT signaling pathway while concurrently dampen the events directed to the ERK signaling cascade. A second possibility is that I'm-Yunity™ (PSP) functions as a growth factor/cytokine decoy to sequester/trap the relevant growth factor/cytokine required for HL-60 cellular function, or bind the pertinent receptor in ways that block further engagement and interaction with their physiological, cognate ligands. This possibility may be supported by structural resemblance existing between the bioactive constituents present in I'm-Yunity™ (PSP) with proteoglycans occurring in the extracellular matrix of cancer cells [98]. As mentioned in the Introduction, a broad spectrum of bioactive molecules, ranging from those with defined structures and functions, e.g., proteases, nucleases, to others with heterogeneous structures and ill-defined activities, e.g., polysaccharo-peptides and polysaccharides, have been identified and isolated from mushrooms [2,4,99,100]. Finally, it is possible that I'm-Yunity™ (PSP) exerts its effect on STAT1 and ERK by disrupting intracellular signaling events, secondary to its internalization that may involve the formation of a complex between the polysaccharo-peptides with specific components in the serum to facilitate its uptake into cells. This mechanism may draw analogy from lipid-mediated delivery of highly charged and heterogeneous plasmid DNA into cultured mammalian cells and as vaccines [101], as well as the intestinal absorption of oral heparin (a heterogeneous, charged mucopolysaccharide) [102-104] or heparan sulfate, e.g., chondroitin sulfate [105-107]. Notably, some of these possibilities can be tested experimentally and are being considered for further investigation in our laboratory.

## Conclusion

Treatment of human leukemia cells by water extracts of I'm-Yunity™ (PSP) causes cell cycle arrest and alterations in the expression of apoptogenic/anti-apoptotic and extracellular signaling regulatory proteins, the net result being a reduction in proliferation and an increase in apoptosis. These findings may be part of the mechanisms that underlie or contribute to its reported clinical attributes and overall health benefits.

## Competing interests

The author(s) declare that they have no competing interests.

## Authors' contributions

TCH carried out tissue culture and flow cytometric studies using the core facilities at the Brander Cancer Institute of New York Medical College. TCH designed and performed the real-time PCR analysis with assistance from a former post-doctoral fellow Xiaohua Lu. PW isolated and purified cytosol from mitochondria and performed immunoblot analysis of the isolated fractions in the revision of the paper. SP performed immunoblot analysis using whole cell lysates in the revision of the paper. TCH and JMW designed the experiments and wrote the paper. All authors read and approved the final manuscript.

## Pre-publication history

The pre-publication history for this paper can be accessed here:



## References

[B1] Yang MM, Chen Z, Kwok JS (1992). The anti-tumor effect of a small polypeptide from Coriolus versicolor (SPCV). Am J Chin Med.

[B2] Wasser SP, Weis AL (1999). Therapeutic effects of substances occurring in higher Basidiomycetes mushrooms: a modern perspective. Crit Rev Immunol.

[B3] Borchers AT, Stern JS, Hackman RM, Keen CL, Gershwin ME (1999). Mushrooms, tumors, and immunity. Proc Soc Exp Biol Med.

[B4] Kidd PM (2000). The use of mushroom glucans and proteoglycans in cancer treatment. Altern Med Rev.

[B5] Hsieh TC, Wu JM (2001). Cell growth and gene modulatory activities of Yunzhi (Windsor Wunxi) from mushroom Trametes versicolor in androgen-dependent and androgen-insensitive human prostate cancer cells. Int J Oncol.

[B6] Chu KK, Ho SS, Chow AH (2002). Coriolus versicolor: a medicinal mushroom with promising immunotherapeutic values. J Clin Pharmacol.

[B7] Chang R (2002). Bioactive polysaccharides from traditional Chinese medicine herbs as anticancer adjuvants. J Altern Complement Med.

[B8] Chang R, White JD (2002). Asian therapies for cancer – coming of age. J Altern Complement Med.

[B9] Zaidman BZ, Yassin M, Mahajna J, Wasser SP (2005). Medicinal mushroom modulators of molecular targets as cancer therapeutics. Appl Microbiol Biotechnol.

[B10] Fullerton SA, Samadi AA, Tortorelis DG, Choudhury MS, Mallouh C, Tazaki H, Konno S (2000). Induction of apoptosis in human prostatic cancer cells with beta-glucan (Maitake mushroom polysaccharide). Mol Urol.

[B11] Ohno N, Miura NN, Nakajima M, Yadomae T (2000). Antitumor 1,3-beta-glucan from cultured fruit body of Sparassis crispa. Biol Pharm Bull.

[B12] Harada T, Miura N, Adachi Y, Nakajima M, Yadomae T, Ohn N (2002). Effect of SCG, 1,3-beta-D-glucan from Sparassis crispa on the hematopoietic response in cyclophosphamide induced leukopenic mice. Biol Pharm Bull.

[B13] Zhang M, Cheung PC, Zhang L (2001). Evaluation of mushroom dietary fiber (nonstarch polysaccharides) from sclerotia of Pleurotus tuber-regium (Fries) singer as a potential antitumor agent. J Agric Food Chem.

[B14] Ohno N, Furukawa M, Miura NN, Adachi Y, Motoi M, Yadomae T (2001). Antitumor beta glucan from the cultured fruit body of Agaricus blazei. Biol Pharm Bull.

[B15] Takaku T, Kimura Y, Okuda H (2001). Isolation of an antitumor compound from Agaricus blazei Murill and its mechanism of action. J Nutr.

[B16] Wang HX, Liu WK, Ng TB, Ooi VE, Chang ST (1995). Immunomodulatory and antitumor activities of a polysaccharide-peptide complex from a mycelial culture of Tricholoma sp., a local edible mushroom. Life Sci.

[B17] Wang H, Gao J, Ng TB (2000). A new lectin with highly potent antihepatoma and antisarcoma activities from the oyster mushroom Pleurotus ostreatus. Biochem Biophys Res Commun.

[B18] Wang HX, Ng TB (2001). Examination of lectins, polysaccharopeptide, polysaccharide, alkaloid, coumarin and trypsin inhibitors for inhibitory activity against human immunodeficiency virus reverse transcriptase and glycohydrolases. Planta Med.

[B19] Mayell M (2001). Maitake extracts and their therapeutic potential. Altern Med Rev.

[B20] Nanba H, Kubo K (1997). Effect of Maitake D-fraction on cancer prevention. Ann N Y Acad Sci.

[B21] Matsui K, Kodama N, Nanba H (2001). Effects of maitake (Grifola frondosa) D-Fraction on the carcinoma angiogenesis. Cancer Lett.

[B22] Finkelstein MP, Aynehchi S, Samadi AA, Drinis S, Choudhury MS, Tazaki H, Konno S (2002). Chemosensitization of carmustine with maitake beta-glucan on androgen-independent prostatic cancer cells: involvement of glyoxalase I. J Altern Complement Med.

[B23] Kawamura Y, Manabe M, Kitta K (2000). Antitumor protein (AP) from a mushroom induced apoptosis to transformed human keratinocyte by controlling the status of prb, c-MYC, cyclin E-cdk2, and p21WAF1 in the G1/S transition. Biofactors.

[B24] Dong Y, Yang MM, Kwan CY (1997). In vitro inhibition of proliferation of HL-60 cells by tetrandrine and coriolus versicolor peptide derived from Chinese medicinal herbs. Life Sci.

[B25] Ng TB (1998). A review of research on the protein-bound polysaccharide (polysaccharopeptide, PSP) from the mushroom Coriolus versicolor (Basidiomycetes: Polyporaceae). Gen Pharmacol.

[B26] Li XY, Wang JF, Zhu PP, Liu L, Ge JB, Yang SX (1990). Immune enhancement of a polysaccharides peptides isolated from Coriolus versicolor. Zhongguo Yao Li Xue Bao.

[B27] Cui J, Chisti Y (2003). Polysaccharopeptides of Coriolus versicolor: physiological activity, uses, and production. Biotechnol Adv.

[B28] Hsieh TC, Kunicki J, Darzynkiewicz Z, Wu JM (2002). Effects of extracts of Coriolus versicolor (I'm-Yunity) on cell-cycle progression and expression of interleukins-1 beta,-6, and -8 in promyelocytic HL-60 leukemic cells and mitogenically stimulated and nonstimulated human lymphocytes. J Altern Complement Med.

[B29] Zeng F, Hon CC, Sit WH, Chow KY, Hui RK, Law IK, Ng VW, Yang XT, Leung FC, Wan JM (2005). Molecular characterization of Coriolus versicolor PSP-induced apoptosis in human promyelotic leukemic HL-60 cells using cDNA microarray. Int J Oncol.

[B30] Hui KP, Sit WH, Wan JM (2005). Induction of S phase cell arrest and caspase activation by polysaccharide peptide isolated from Coriolus versicolor enhanced the cell cycle dependent activity and apoptotic cell death of doxorubicin and etoposide, but not cytarabine in HL-60 cells. Oncol Rep.

[B31] Yang X, Sit WH, Chan DK, Wan JM (2005). The cell death process of the anticancer agent polysaccharide-peptide (PSP) in human promyelocytic leukemic HL-60 cells. Oncol Rep.

[B32] Dipietrantonio A, Hsieh TC, Wu JM (1996). Differential effects of retinoic acid (RA) and N-(4-hydroxyphenyl) retinamide (4-HPR) on cell growth, induction of differentiation, and changes in p34cdc2, Bcl-2, and actin expression in the human promyelocytic HL-60 leukemic cells. Biochem Biophys Res Commun.

[B33] DiPietrantonio AM, Hsieh TC, Olson SC, Wu JM (1998). Regulation of G1/S transition and induction of apoptosis in HL-60 leukemia cells by fenretinide (4HPR). Int J Cancer.

[B34] Hsieh TC, Wu JM (2002). Mechanism of action of herbal supplement PC-SPES: elucidation of effects of individual herbs of PC-SPES on proliferation and prostate specific gene expression in androgen-dependent LNCaP cells. Int J Oncol.

[B35] Hsieh TC, Wang Z, Hamby CV, Wu JM (2005). Inhibition of melanoma cell proliferation by resveratrol is correlated with upregulation of quinone reductase 2 and p53. Biochem Biophys Res Commun.

[B36] Hsieh TC, Wu JM (1999). Differential effects on growth, cell cycle arrest, and induction of apoptosis by resveratrol in human prostate cancer cell lines. Exp Cell Res.

[B37] Darzynkiewicz Z, Bedner E, Smolewski P (2001). Flow cytometry in analysis of cell cycle and apoptosis. Semin Hematol.

[B38] Fu Y, Hsieh TC, Guo J, Kunicki J, Lee MY, Darzynkiewicz Z, Wu JM (2004). Licochalcone-A, a novel flavonoid isolated from licorice root (Glycyrrhiza glabra), causes G2 and late-G1 arrests in androgen-independent PC-3 prostate cancer cells. Biochem Biophys Res Commun.

[B39] Hsieh TC, Juan G, Darzynkiewicz Z, Wu JM (1999). Resveratrol increases nitric oxide synthase, induces accumulation of p53 and p21(WAF1/CIP1), and suppresses cultured bovine pulmonary artery endothelial cell proliferation by perturbing progression through S and G2. Cancer Res.

[B40] Lu X, Hsieh TC, Wu JM (2004). Equiguard suppresses androgen-dependent LNCaP prostate cancer cell proliferation by targeting cell cycle control via down regulation of the retinoblastoma protein Rb and induction of apoptosis via the release of cytochrome c. Int J Oncol.

[B41] Lu X, Guo J, Hsieh TC (2003). PC-SPES inhibits cell proliferation by modulating p21, cyclins D, E and B and multiple cell cycle-related genes in prostate cancer cells. Cell Cycle.

[B42] Hsieh TC, Lu X, Guo J, Xiong W, Kunicki J, Darzynkiewicz Z, Wu JM (2002). Effects of herbal preparation Equiguard on hormone-responsive and hormone-refractory prostate carcinoma cells: mechanistic studies. Int J Oncol.

[B43] Lu X, Guo J, Hsieh TC, Wu JM (2003). Inhibition of proliferation and expression of AR/PSA by herbal supplement Equiguard in LNCaP cells cultured in androgen-proficient FBS and androgen-deficient charcoal-stripped FBS is correlated with increased serine-15 phosphorylation of the tumor suppressor gene p53. Anticancer Res.

[B44] Taya Y (1997). RB kinases and RB-binding proteins: new points of view. Trends Biochem Sci.

[B45] Darzynkiewicz Z, Bedner E, Traganos F, Murakami T (1998). Critical aspects in the analysis of apoptosis and necrosis. Hum Cell.

[B46] Wu JM, DiPietrantonio AM, Hsieh TC (2001). Mechanism of fenretinide (4-HPR)-induced cell death. Apoptosis.

[B47] Darzynkiewicz Z, Smolewski P, Bedner E (2001). Use of flow and laser scanning cytometry to study mechanisms regulating cell cycle and controlling cell death. Clin Lab Med.

[B48] Darzynkiewicz Z, Huang X, Okafuji M, King MA (2004). Cytometric methods to detect apoptosis. Methods Cell Biol.

[B49] Thompson CB (1995). Apoptosis in the pathogenesis and treatment of disease. Science.

[B50] Duckett CS, Thompson CB (1997). The control and execution of programmed cell death: an update. Biochim Biophys Acta.

[B51] Tsai SY, Ardelt B, Hsieh TC, Darzynkiewicz Z, Shogen K, Wu JM (2004). Treatment of Jurkat acute T-lymphocytic leukemia cells by onconase (Ranpirnase) is accompanied by an altered nucleocytoplasmic distribution and reduced expression of transcription factor NF-kappaB. Int J Oncol.

[B52] Perkins ND, Gilmore TD (2006). Good cop, bad cop: the different faces of NF-kappaB. Cell Death Differ.

[B53] Campbell KJ, Perkins ND (2006). Regulation of NF-kappaB function. Biochem Soc Symp.

[B54] Aggarwal BB (2004). Nuclear factor-kappaB: the enemy within. Cancer Cell.

[B55] Shishodia S, Aggarwal BB (2004). Nuclear factor-kappaB: a friend or a foe in cancer?. Biochem Pharmacol.

[B56] Kumar A, Takada Y, Boriek AM, Aggarwal BB (2004). Nuclear factor-kappaB: its role in health and disease. J Mol Med.

[B57] Shishodia S, Aggarwal BB (2004). Nuclear factor-kappaB activation mediates cellular transformation, proliferation, invasion angiogenesis and metastasis of cancer. Cancer Treat Res.

[B58] Horvath CM (2004). The Jak-STAT pathway stimulated by interferon gamma. Sci STKE.

[B59] Qing Y, Stark GR (2004). Alternative activation of STAT1 and STAT3 in response to interferon-gamma. J Biol Chem.

[B60] Kerr IM, Costa-Pereira AP, Lillemeier BF, Strobl B (2003). Of JAKs, STATs, blind watchmakers, jeeps and trains. FEBS Lett.

[B61] Sehgal PB, Guo GG, Shah M, Kumar V, Patel K (2002). Cytokine signaling: STATS in plasma membrane rafts. J Biol Chem.

[B62] Shah M, Patel K, Fried VA, Sehgal PB (2002). Interactions of STAT3 with caveolin-1 and heat shock protein 90 in plasma membrane raft and cytosolic complexes. Preservation of cytokine signaling during fever. J Biol Chem.

[B63] Lee CK, Smith E, Gimeno R, Gertner R, Levy DE (2000). STAT1 affects lymphocyte survival and proliferation partially independent of its role downstream of IFN-gamma. J Immunol.

[B64] Doll R, Peto R (1981). The causes of cancer: quantitative estimates of avoidable risks of cancer in the United States today. J Natl Cancer Inst.

[B65] Hsing AW, Tsao L, Devesa SS (2000). International trends and patterns of prostate cancer incidence and mortality. Int J Cancer.

[B66] Hsing AW, Devesa SS (2001). Trends and patterns of prostate cancer: what do they suggest?. Epidemiol Rev.

[B67] Sporn MB, Suh N (2002). Chemoprevention: an essential approach to controlling cancer. Nat Rev Cancer.

[B68] Sporn MB, Newton DL (1979). Chemoprevention of cancer with retinoids. Fed Proc.

[B69] Sporn MB, Suh N (2000). Chemoprevention of cancer. Carcinogenesis.

[B70] Milner JA, McDonald SS, Anderson DE, Greenwald P (2001). Molecular targets for nutrients involved with cancer prevention. Nutr Cancer.

[B71] Greenwald P (2002). Cancer prevention clinical trials. J Clin Oncol.

[B72] Greenwald P (2002). Cancer chemoprevention. Bmj.

[B73] Greenwald P (2004). Clinical trials in cancer prevention: current results and perspectives for the future. J Nutr.

[B74] Greenwald P, Clifford CK, Milner JA (2001). Diet and cancer prevention. Eur J Cancer.

[B75] Greenwald P, McDonald SS, Anderson DE (2002). An evidence-based approach to cancer prevention clinical trials. Eur J Cancer Prev.

[B76] Greenwald P, Milner JA, Anderson DE, McDonald SS (2002). Micronutrients in cancer chemoprevention. Cancer Metastasis Rev.

[B77] Hong WK, Sporn MB (1997). Recent advances in chemoprevention of cancer. Science.

[B78] Kelloff GJ, Hawk ET, Karp JE, Crowell JA, Boone CW, Steele VE, Lubet RA, Sigman CC (1997). Progress in clinical chemoprevention. Semin Oncol.

[B79] Kelloff GJ, Lieberman R, Steele VE, Boone CW, Lubet RA, Kopelovich L, Malone WA, Crowell JA, Higley HR, Sigman CC (2001). Agents, biomarkers, and cohorts for chemopreventive agent development in prostate cancer. Urology.

[B80] Perkins ND (2004). NF-kappaB: tumor promoter or suppressor?. Trends Cell Biol.

[B81] Campbell KJ, O'Shea JM, Perkins ND (2006). Differential regulation of NF-kappaB activation and function by topoisomerase II inhibitors. BMC Cancer.

[B82] Campbell KJ, Witty JM, Rocha S, Perkins ND (2006). Cisplatin mimics ARF tumor suppressor regulation of RelA (p65) nuclear factor-kappaB transactivation. Cancer Res.

[B83] Gimonet D, Landais E, Bobichon H, Coninx P, Liautaud-Roger F (2004). Induction of apoptosis by bleomycin in p53-null HL-60 leukemia cells. Int J Oncol.

[B84] Shimizu T, Pommier Y (1996). DNA fragmentation induced by protease activation in p53-null human leukemia HL60 cells undergoing apoptosis following treatment with the topoisomerase I inhibitor camptothecin: cell-free system studies. Exp Cell Res.

[B85] El-Mahdy MA, Zhu Q, Wang QE, Wani G, Wani AA (2005). Thymoquinone induces apoptosis through activation of caspase-8 and mitochondrial events in p53-null myeloblastic leukemia HL-60 cells. Int J Cancer.

[B86] Houle MG, Kahn RA, Naccache PH, Bourgoin S (1995). ADP-ribosylation factor translocation correlates with potentiation of GTP gamma S-stimulated phospholipase D activity in membrane fractions of HL-60 cells. J Biol Chem.

[B87] Martin A, Brown FD, Hodgkin MN, Bradwell AJ, Cook SJ, Hart M, Wakelam MJ (1996). Activation of phospholipase D and phosphatidylinositol 4-phosphate 5-kinase in HL60 membranes is mediated by endogenous Arf but not Rho. J Biol Chem.

[B88] Guillemain I, Exton JH (1997). Effects of brefeldin A on phosphatidylcholine phospholipase D and inositolphospholipid metabolism in HL-60 cells. Eur J Biochem.

[B89] Skippen A, Jones DH, Morgan CP, Li M, Cockcroft S (2002). Mechanism of ADP ribosylation factor-stimulated phosphatidylinositol 4,5-bisphosphate synthesis in HL60 cells. J Biol Chem.

[B90] Denk A, Goebeler M, Schmid S, Berberich I, Ritz O, Lindemann D, Ludwig S, Wirth T (2001). Activation of NF-kappa B via the Ikappa B kinase complex is both essential and sufficient for proinflammatory gene expression in primary endothelial cells. J Biol Chem.

[B91] Perona R, Montaner S, Saniger L, Sanchez-Perez I, Bravo R, Lacal JC (1997). Activation of the nuclear factor-kappaB by Rho, CDC42, and Rac-1 proteins. Genes Dev.

[B92] Nelson WG, De Marzo AM, Isaacs WB (2003). Prostate cancer. N Engl J Med.

[B93] Narayanan BA, Narayanan NK, Pittman B, Reddy BS (2004). Regression of mouse prostatic intraepithelial neoplasia by nonsteroidal anti-inflammatory drugs in the transgenic adenocarcinoma mouse prostate model. Clin Cancer Res.

[B94] Narayanan BA, Narayanan NK, Pttman B, Reddy BS (2005). Adenocarcina of the mouse prostate growth inhibition by celecoxib: Downregulation of transcription factors involved in COX-2 inhibition. Prostate.

[B95] Kashfi K, Rigas B (2005). Is COX-2 a 'collateral' target in cancer prevention?. Biochem Soc Trans.

[B96] Ranger GS, Thomas V, Jewell A, Mokbel K (2004). Elevated cyclooxygenase-2 expression correlates with distant metastases in breast cancer. Anticancer Res.

[B97] Spano JP, Chouahnia K, Morere JF (2004). [Cyclooxygenase 2 inhibitors and lung carcinoma]. Bull Cancer.

[B98] Monro JA (2003). Treatment of cancer with mushroom products. Arch Environ Health.

[B99] Wasser SP (2002). Medicinal mushrooms as a source of antitumor and immunomodulating polysaccharides. Appl Microbiol Biotechnol.

[B100] Fisher M, Yang LX (2002). Anticancer effects and mechanisms of polysaccharide-K (PSK): implications of cancer immunotherapy. Anticancer Res.

[B101] Howard KA, Li XW, Somavarapu S, Singh J, Green N, Atuah KN, Ozsoy Y, Seymour LW, Alpar HO (2004). Formulation of a microparticle carrier for oral polyplex-based DNA vaccines. Biochim Biophys Acta.

[B102] Ross BP, Toth I (2005). Gastrointestinal absorption of heparin by lipidization or coadministration with penetration enhancers. Curr Drug Deliv.

[B103] Kim SK, Lee EH, Vaishali B, Lee S, Lee YK, Kim CY, Moon HT, Byun Y (2005). Tricaprylin microemulsion for oral delivery of low molecular weight heparin conjugates. J Control Release.

[B104] Kim SK, Vaishali B, Lee E, Lee S, Lee YK, Kumar TS, Moon HT, Byun Y (2005). Oral delivery of chemical conjugates of heparin and deoxycholic acid in aqueous formulation. Thromb Res.

[B105] Bernkop-Schnurch A, Kast CE, Guggi D (2003). Permeation enhancing polymers in oral delivery of hydrophilic macromolecules: thiomer/GSH systems. J Control Release.

[B106] Tsai MF, Chiang YL, Wang LF, Huang GW, Wu PC (2005). Oral sustained delivery of diclofenac sodium using calcium chondroitin sulfate matrix. J Biomater Sci Polym Ed.

[B107] Sinha VR, Kumria R (2001). Polysaccharides in colon-specific drug delivery. Int J Pharm.

